# Evaluation of Toxicity Equivalency Factors of Tetrodotoxin Analogues with a Neuro-2a Cell-Based Assay and Application to Puffer Fish from Greece

**DOI:** 10.3390/md21080432

**Published:** 2023-07-29

**Authors:** Mounira Alkassar, Andres Sanchez-Henao, Jaume Reverté, Lourdes Barreiro, Maria Rambla-Alegre, Sandra Leonardo, Manolis Mandalakis, Panagiota Peristeraki, Jorge Diogène, Mònica Campàs

**Affiliations:** 1 Institut de Recerca i Tecnologia Agroalimentàries (IRTA), Ctra. Poble Nou km 5.5, 43540 La Ràpita, Spain; mounira.alkassar@irta.cat (M.A.); julian.sanchez@irta.cat (A.S.-H.); jaume.reverte@irta.cat (J.R.); lourdes.barreiro@irta.cat (L.B.); maria.rambla@irta.cat (M.R.-A.); sandra.leonardo@irta.cat (S.L.); jorge.diogene@irta.cat (J.D.); 2 Institute of Marine Biology, Biotechnology and Aquaculture, Hellenic Centre for Marine Research, 71003 Heraklion, Greece; mandalakis@hcmr.gr; 3 Institute of Marine Biological Resources and Inland Waters, Hellenic Centre for Marine Research, 71003 Heraklion, Greece; notap@hcmr.gr

**Keywords:** tetrodotoxin (TTX), TTX analogue, toxicity equivalency factor (TEF), cell-based assay (CBA), liquid chromatography-tandem mass spectrometry (LC-MS/MS), puffer fish, *Lagocephalus sceleratus*

## Abstract

Tetrodotoxin (TTX) is a potent marine neurotoxin involved in poisoning cases, especially through the consumption of puffer fish. Knowledge of the toxicity equivalency factors (TEFs) of TTX analogues is crucial in monitoring programs to estimate the toxicity of samples analyzed with instrumental analysis methods. In this work, TTX analogues were isolated from the liver of a *Lagocephalus sceleratus* individual caught on South Crete coasts. A cell-based assay (CBA) for TTXs was optimized and applied to the establishment of the TEFs of 5,11-dideoxyTTX, 11-norTTX-6(*S*)-ol, 11-deoxyTTX and 5,6,11-trideoxyTTX. Results showed that all TTX analogues were less toxic than the parent TTX, their TEFs being in the range of 0.75–0.011. Then, different tissues of three *Lagocephalus sceleratus* individuals were analyzed with CBA and liquid chromatography-tandem mass spectrometry (LC-MS/MS). The obtained TEFs were applied to the TTX analogues’ concentrations obtained by LC-MS/MS analysis, providing an indication of the overall toxicity of the sample. Information about the TEFs of TTX analogues is valuable for food safety control, allowing the estimation of the risk of fish products to consumers.

## 1. Introduction

Tetrodotoxin (TTX) is a water-soluble molecule associated with neurotoxic marine poisonings [1]. Structurally, TTX (C_11_H_17_O_8_N_3_) has a low molecular weight (319.27 g/mol) and consists of a guanidinium moiety connected to a highly oxygenated carbon backbone with a 2,4-dioxaadamantane portion containing five hydroxyl groups [2]. It was initially thought to be present only in pufferfish [3]; however, it has been later reported in a wide range of marine and terrestrial animals [4,5,6,7]. Many bacteria, such as *Pseudomonas* sp., *Vibrio* sp., and *Alteromonas* sp., have been observed to produce TTX, but the contribution of micro-organisms to TTX bioaccumulation in marine ecosystems is still not fully elucidated [8,9]. To date, more than 30 analogues of TTX have been described, with varying toxic potencies, many of which are detected in puffer fishes [10]. TTX and its analogues are known to selectively block the voltage-gated sodium channels (VSGCs). This blocking inhibits the entry of sodium ions into the nerve cells through the cell membranes, leading to acute health effects in animals and humans [11].

Tetrodotoxin intoxications have been reported in many Asian countries, especially in Japan, where puffer fish is consumed and considered as a delicacy [12]. However, in the last few years, tetrodotoxication incidents have been encountered in a larger geographical area, and around 156 poisoning cases have been reported in several Mediterranean countries, which have been mainly attributed to *L. sceleratus* [13,14,15,16,17,18,19,20,21]. Regarding regulations, the Japanese government has established a regulatory limit of 2 mg TTX equiv./kg of tissue in puffer fish [22]. Within Europe, there is legislation prohibiting the trade of fish belonging to the *Tetraodontidae* family or products derived from them [23]. It is important to mention that, recently, TTX has also been found in shellfish from European countries [24,25,26,27]. The CONTAM panel of the European Food Safety Authority (EFSA) concluded that a concentration lower than 44 μg of TTX and/or the equivalent toxic amount of its analogues per kg of shellfish meat is not expected to result in adverse effects in humans [28]. They also recommend exploring the possibility of combining STX and its analogues together with TTX and its analogues in one health-based guidance value, taking into account the fact that they exert similar toxic effects via a similar mode of action, and their toxicities are additive [29].

Tetrodotoxin analysis has been performed using several detection methods throughout the years [30]. Traditionally, the mouse bioassay (MBA) has been the most widely applied test for the assessment of TTX toxicity in puffer fish. However, this biological method is not suitable for obtaining quantitative data and suffers from low specificity and ethical concerns. As an alternative to the MBA, cell-based assays (CBAs) for TTX detection have been described [31]. These assays are very useful as screening tools, since they are based on the mechanism of action of TTX and, therefore, they also provide a composite toxicological response. The application of CBA for TTX requires the use of veratridine (V), a Na^+^ channel activator, and ouabain (O), a Na^+^/K^+^-ATPase inhibitor. The main role of these compounds is to increase sodium ions into the cells, causing their mortality. TTX and its analogues counteract the effect of V and O, increasing the viability of cells in a dose-dependent manner. Immunochemical assays and biosensors have recently emerged as new tools for the detection of TTX in natural samples [13,32,33,34,35,36]. They also provide a composite response, but based on structural information, and, therefore, not related to the toxicity of a sample [37,38]. Instrumental analysis techniques, such as LC-MS/MS, have also been widely used for TTX analysis in puffer fish samples, as TTX and its analogues can be identified and quantified with high accuracy and specificity [15,16,17,18,19,20,21,39,40]. Although these techniques provide information about the toxin profile, they do not provide information about the toxicity of a sample.

For risk assessment purposes, the total toxicity of biological samples can be estimated using LC-MS/MS quantification data of the different TTX analogues, but this approach requires the determination of toxicity equivalency factors (TEFs). These provide the relative toxicity of an analogue in relation to the parent toxin, in this case, TTX. The use of TEFs is very important in monitoring programs aiming to assess the toxicity of samples and guarantee that toxin levels are below the limits defined in relevant legislation. However, the information available on the relative toxicity of TTX analogues is scarce, mainly due to the lack of commercially available standards. In fact, the Food and Agriculture Organization and the World Health Organization (FAO/WHO) set up an expert group to develop a technical paper providing scientific explanations and recommendations on TEFs, which noted the need to establish TEFs for TTX [41]. They also agreed on an order of priority: data from human intoxications (the most relevant but very scarce), acute toxicity data through oral administration to animals (relevant but with ethical concerns), acute toxicity data through intraperitoneal administration to animals (less valuable and also with ethical concerns), and in vitro data, such as CBA (particularly useful) [42].

The present study aimed at elucidating the TEFs of four TTX analogues (5,11-dideoxyTTX, 11-norTTX-6(*S*)-ol, 11-deoxyTTX, and 5,6,11-trideoxyTTX) using a CBA approach. Firstly, the concentration and ratio of O and V were optimized for TTX detection. Then, the CBA was applied to detect TTXs in different tissues of three *L. sceleratus* specimens caught in the Libyan Sea (South Crete, Greece, Eastern Mediterranean Sea). The characterization of the toxin profile of these tissues was assessed using LC-MS/MS analysis. After the purification and isolation of four TTX analogues from an *L. sceleratus* liver extract, the optimized CBA was applied to establish the TEFs of the specific analogues. The derived TEFs were then applied to the concentrations of the different analogues measured in *L. sceleratus* samples by LC-MS/MS to estimate total toxicity expressed in TTX equivalents.

## 2. Results

### 2.1. Cell-Based Assay (CBA) for Tetrodotoxin (TTX)

As previously mentioned, the detection of TTX with CBA requires the addition of O and V, which induce cytotoxicity. Tetrodotoxin counteracts the toxic effect of O/V in a concentration-dependent manner, resulting in higher cell viability. Therefore, the first step in the development of the CBA for TTXs was the optimization of the O and V concentrations and their ratio. To find the optimal O/V conditions, different experimental parameters were considered. First, the viability of cells exposed to O/V was evaluated. Ideally, O/V conditions have to be set so that a cell viability of approximately 20% is attained. This viability of cells exposed to O/V was expressed as a percentage of the following absorbance (ABS) values:

$$\%\;\mathrm{cell}\;\mathrm{viability}=\frac{\mathrm{ABS}\;\mathrm{from}\;\mathrm{cells}\;\mathrm{exposed}\;\mathrm{to}\;O/V}{\mathrm{ABS}\;\mathrm{from}\;\mathrm{control}\;\mathrm{cells}\;\mathrm{without}\;O/V\;\mathrm{pre}-\mathrm{treatment}}\times 100$$

Then, calibration curves for TTX in the presence of different O/V combinations were constructed. In this case, the viability of cells exposed to TTX and O/V was expressed as a percentage of the following ABS values:

$$\%\;\mathrm{cell}\;\mathrm{viability}=\frac{\mathrm{ABS}\;\mathrm{from}\;\mathrm{cells}\;\mathrm{exposed}\;\mathrm{to}\;\mathrm{TTX}\;\mathrm{and}\;O/V-\mathrm{ABS}\;\mathrm{from}\;\mathrm{cells}\;\mathrm{exposed}\;\mathrm{to}\;O/V}{\mathrm{ABS}\;\mathrm{from}\;\mathrm{control}\;\mathrm{cells}\;\mathrm{without}\;O/V\;\mathrm{pre}-\mathrm{treatment}-\mathrm{ABS}\;\mathrm{from}\;\mathrm{cells}\;\mathrm{exposed}\;\mathrm{to}\;O/V}\times 100$$

**Figure 1:**
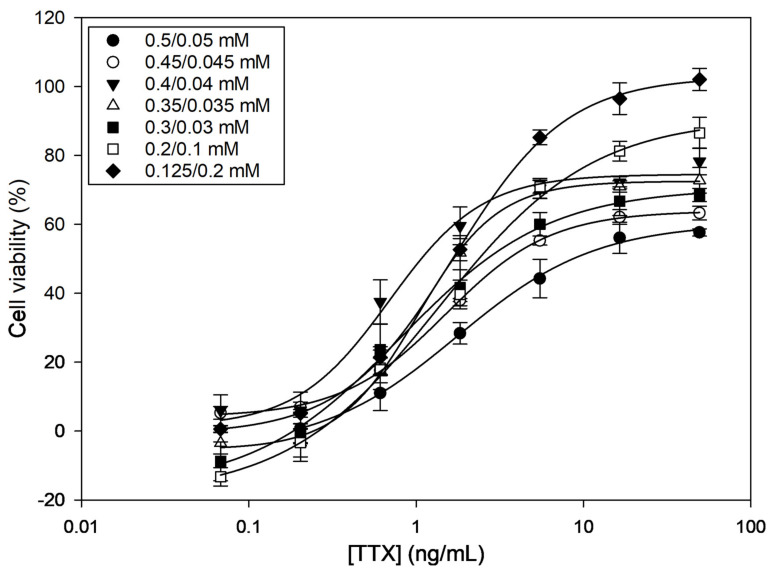
Dose–response curves for tetrodotoxin (TTX) in presence of different ouabain (O)/veratridine (V) combinations as determined by Neuro-2a cell-based assay (CBA). Each point shows the average and standard deviation of three replicates.

Among the different combinations tested, the O/V concentration ratios 0.4/0.04, 0.35/0.035, and 0.125/0.2 (in mM) caused around 80% mortality (i.e., 20% viability) (Table 1). All other O/V combinations caused lower mortality. When exposing cells to different TTX concentrations, dose–response curves were obtained for all O/V combinations (Figure 1), indicating that TTX counteracts the effect of O/V. However, curves showed different half-maximal inhibitory concentration (IC_50_) values and saturation plateaus. The three O/V combinations that caused the highest mortalities were also the ones that provided the lowest IC_50_ values (Table 1). However, 0.4/0.04 and 0.35/0.035 O/V combinations only reached around ~70% of cell viability at the highest TTX concentrations. Therefore, the 0.125/0.2 O/V combination, which reached ~100% cell viability and, therefore, showed a wider working range, was chosen as the optimal experimental condition.

### 2.2. Isolation and Purification of Tetrodotoxin (TTX) Analogues

**Figure 2:**
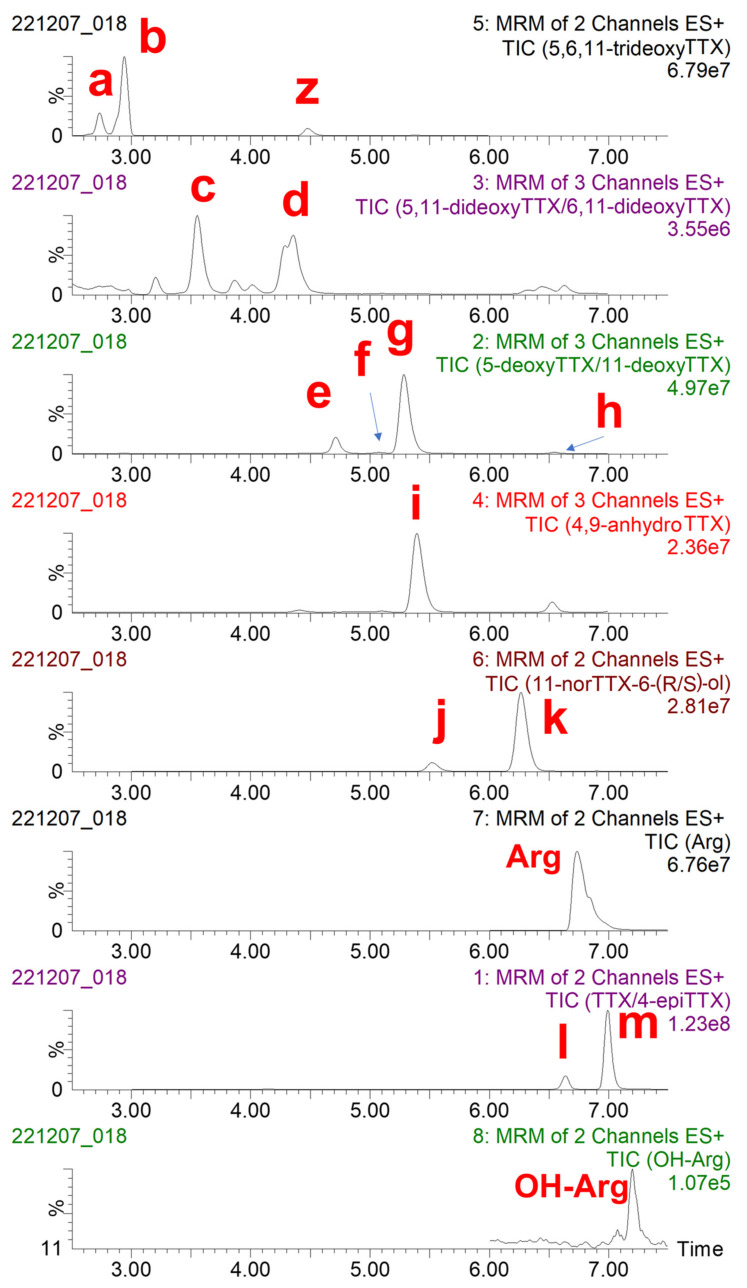
Total ion chromatogram (TIC) of transitions monitored, obtained following the analysis of TTX and TTX analogues in the concentrated PF2 liver extract by liquid chromatography-tandem mass spectrometry (LC-MS/MS) analysis. (a) 4-epi-5,6,11-trideoxyTTX, (b) 5,6,11-trideoxyTTX, (c) 5,11-dideoxyTTX, (d) 6,11-dideoxyTTX, (e) 5-deoxyTTX, (f) 4-epi-11-deoxyTTX, (g) 11-deoxyTTX, (h) 6-epi-11-deoxyTTX, (i) 4,9-anydroTTX, (j) 11-norTTX-6-(R)-ol, (k) 11-norTTX-6-(S)-ol, (l) 4-epiTTX, and (m) TTX. Arg: arginine, OH-Arg: hydroxyarginine, z: interfering substance.

Initially, the toxin profile of the concentrated PF2 liver extract was characterized by LC-MS/MS (Figure 2). Each chromatogram recorded was analyzed to identify TTX, known TTX analogues, and the possible uncommon/unknown TTX analogues. Additionally, the aminoacid arginine (Arg) and hydroxyarginine (OH-Arg) were monitored as described elsewhere [43] because these have been found to suppress TTX responses in the mass spectrometer source. Some coelutions were observed: 6,11-dideoxyTTX was coeluting with an interfering substance (“z” in Figure 2) and 5-deoxyTTX; 5-deoxyTTX was also coeluting with 4-*epi*-11-deoxyTTX and 11-deoxyTTX; and 4,9-anydroTTX was coeluting with 11-deoxyTTX and 11-norTTX-6-(*R*)-ol. It should be mentioned that this concentrated PF2 liver extract contained at least 13 TTX analogues, which increases the difficulty of the isolation of the TTX congeners.

**Figure A1:**
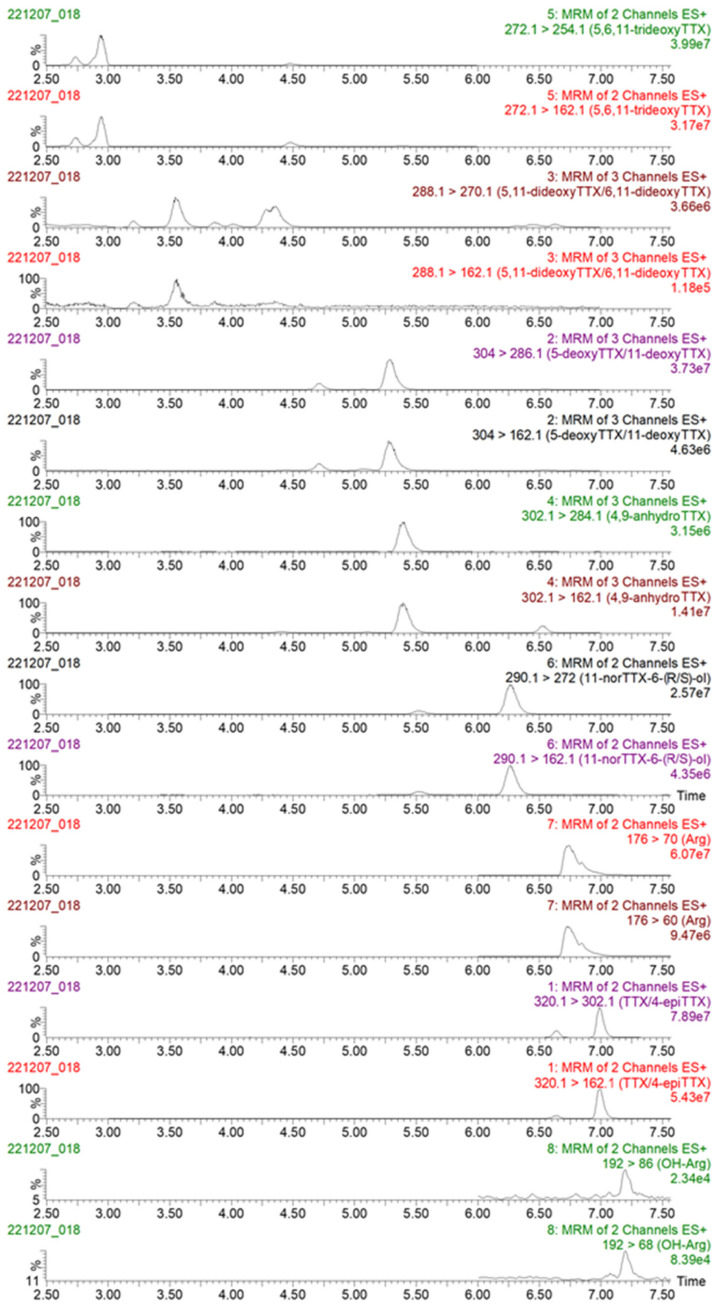
Multiple reaction monitoring (MRM) chromatograms of transitions monitored, obtained following the analysis of TTX and its analogues in the concentrated PF2 liver extract by LC-MS/MS.

The preparative chromatographic method was then applied to the concentrated PF2 liver extract in order to obtain the different TTX analogues. The previously mentioned coelutions were also observed. Nevertheless, the different TTX analogues could be quantified because of their specific multiple reaction monitoring (MRM) transitions (Figure A1). Fractionation allowed us to isolate 5,11-dideoxyTTX, 11-norTTX-6(*S*)-ol, 11-deoxyTTX, and 5,6,11-trideoxyTTX with different grades of purity (Table A1). The purity of TTX analogues in the fractions was calculated as the percentage of the major TTX analogue with respect to the total amount of all TTX analogues found in that fraction, and it was 93%, 98%, 94%, and 85% for 5,6,11-trideoxyTTX (fraction 23), 5,11-dideoxyTTX (fractions 31 and 32), 11-deoxyTTX (fraction 49), and 11-norTTX-6(*S*)-ol (fraction 57), respectively. It was not possible to isolate some other analogues with a purity higher than 85%.

### 2.3. Toxicity Equivalency Factors (TEFs) of Tetrodotoxin (TTX) Analogues

**Figure 3:**
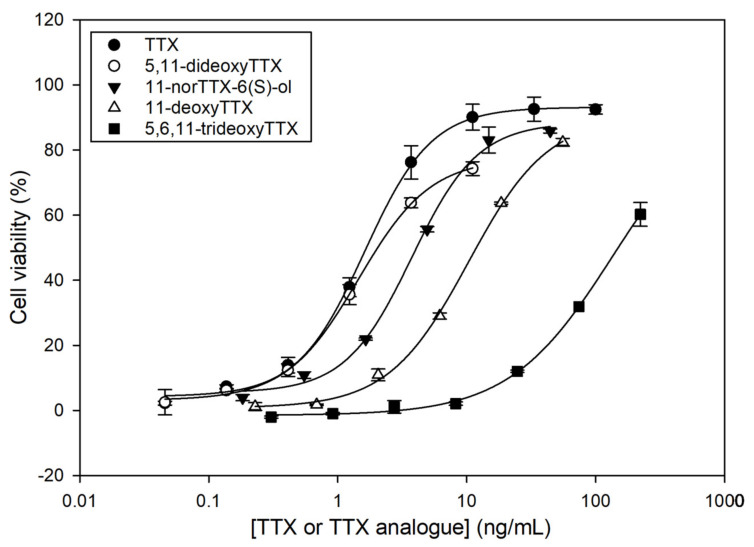
Dose–response curves for tetrodotoxin (TTX) and TTX analogues as determined by Neuro-2a cell-based assay (CBA). Each point shows the average and standard deviation of three replicates.

In order to elucidate the TEFs of the TTX analogues obtained in the fractionation process, calibration curves for TTX, 5,11-dideoxyTTX, 11-norTTX-6(*S*)-ol, 11-deoxyTTX, and 5,6,11-trideoxyTTX with CBA were constructed. Calibration curves were fitted to sigmoidal logistic four-parameter equations using SigmaPlot 12.0 (Systat Software, San Jose, CA, USA, Figure 3). It is important to explain that Neuro-2a cells with and without O/V pre-treatment were exposed to higher TTX analogue concentrations than those observed in the calibration curves. Although the fractions were dried and reconstituted in culture medium, cells without O/V pre-treatment showed mortality at high TTX analogue concentrations, certainly due to the effect of the chemical solvents used in the fractionation. Therefore, those TTX analogue concentrations showing solvent effects were removed from the calibration curves.

All TTX analogues counteracted the toxic effect of O/V on Neuro-2a cells, resulting in higher cell viability, but less than the parent TTX. The toxicity trend was as follows: TTX > 5,11-dideoxyTTX > 11-norTTX-6(*S*)-ol > 11-deoxyTTX > 5,6,11-trideoxyTTX. The TEF of each TTX analogue was calculated as the ratio of the IC_50_ value of the TTX standard to the IC_50_ value of each TTX analogue (Table 2).

### 2.4. Analysis of Puffer Fish Samples with Cell-Based Assay (CBA)

**Figure 4:**
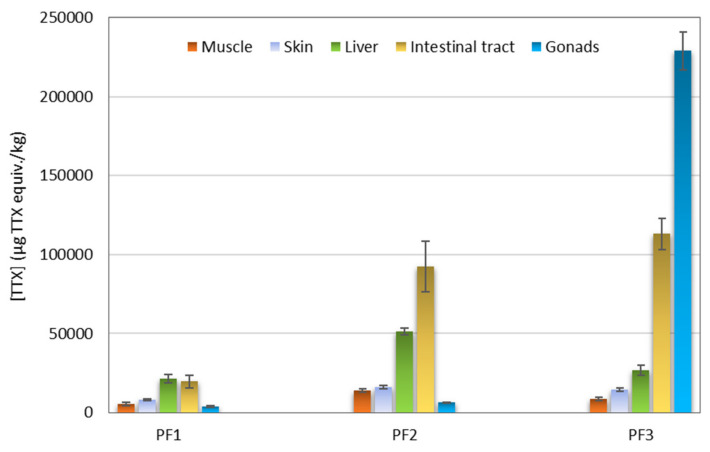
Tetrodotoxin (TTX) equiv. concentration (μg TTX equiv./kg) in different tissues of three puffer fish (*L. sceleratus*) individuals as determined by Neuro-2a cell-based assay (CBA).

The extracts obtained from five tissues (muscle, skin, liver, intestinal tract, and gonads) of three puffer fish individuals (PF1, PF2, and PF3) (Table 3) were analyzed with CBA. The concentrations of TTXs in μg TTX equiv./kg puffer fish tissue are presented in Figure 4. All samples showed TTX equiv. contents higher than the Japanese official regulatory limit of 2 mg/kg. In general terms, PF1 showed the lowest TTX equiv. contents and PF3 the highest ones. With regard to tissue type, intestinal tracts and livers showed higher TTX equiv. contents than skins and muscles. The results from gonads were highly variable with the male specimens PF1 and PF2 showing quite low TTX equiv. contents (~3700 and ~6400 μg TTX equiv./kg, respectively), whereas the gonads of the female specimen PF3 was the most toxic among all the samples analyzed (~229,000 μg TTX equiv./kg).

### 2.5. Analysis of Puffer Fish Samples with Liquid Chromatography-Tandem Mass Spectrometry (LC-MS/MS)

**Figure 5:**
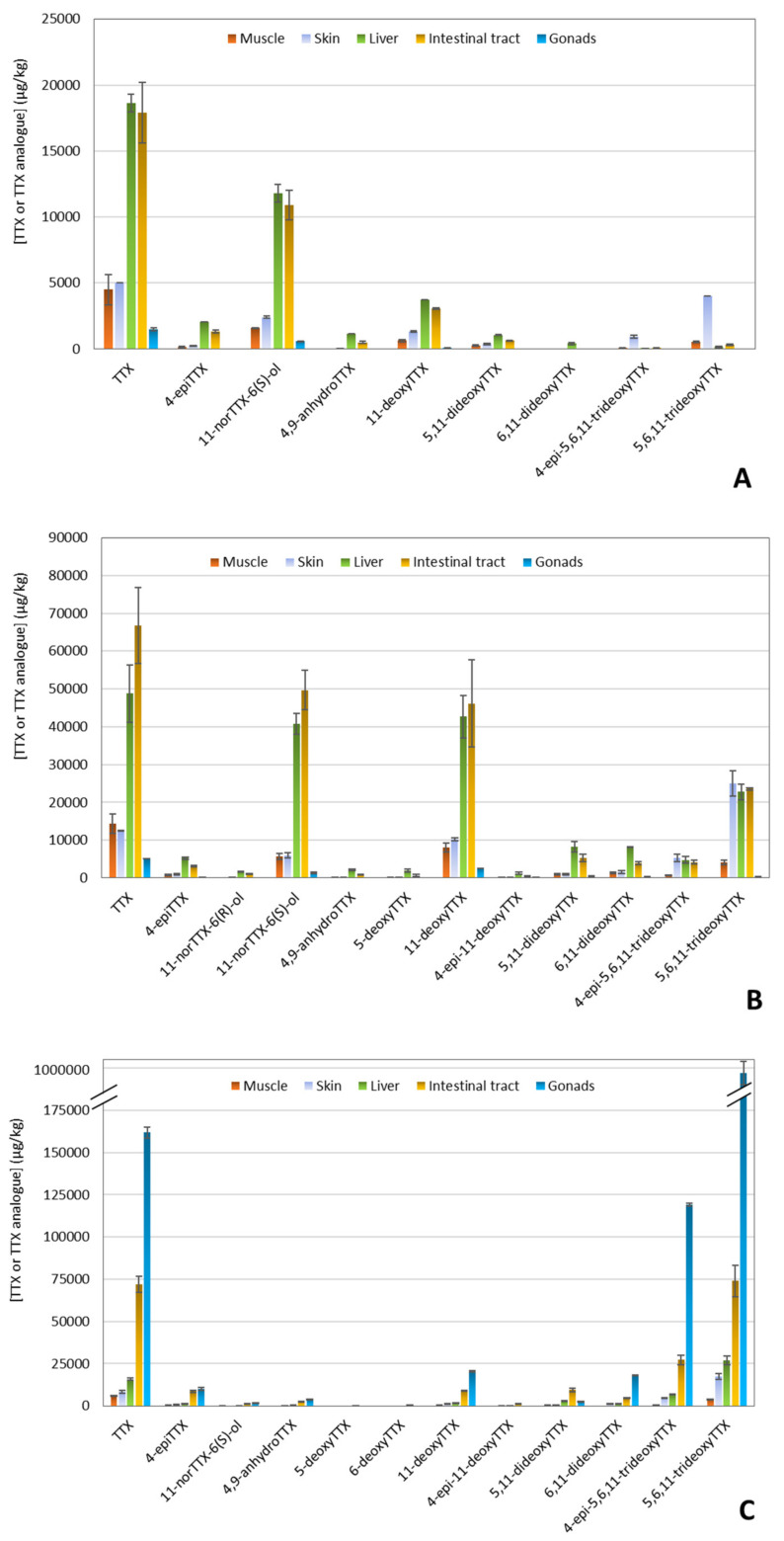
Distribution of tetrodotoxin (TTX) and TTX analogues in different tissues of three puffer fish (*L. sceleratus*) individuals as determined by liquid chromatography-tandem mass spectrometry (LC-MS/MS) analysis. (**A**) Puffer fish 1, (**B**) Puffer fish 2, and (**C**) Puffer fish 3.

To confirm the presence of TTX and to evaluate the presence of TTX analogues in the five tissue extracts of the three puffer fish individuals previously analyzed by CBA, LC-MS/MS analysis was performed. Results of TTX contents among the tissue extracts are summarized in Figure 5. The LC-MS/MS analysis revealed a multi-toxin profile in all samples, characterized by the consistent detection of TTX, but also by the varying presence of some TTX analogues (4-*epi*TTX, 11-norTTX-6(*R*)-ol, 11-norTTX-6(*S*)-ol, 4,9-anhydroTTX, 5-deoxyTTX, 6-deoxyTTX, 11-deoxyTTX, 4-*epi*-11-deoxyTTX, 5,11-dideoxyTTX, 6,11-dideoxyTTX, 5,6,11-trideoxyTTX, and 4-*epi*-5,6,11-trideoxyTTX). In almost all tissues, TTX was found to be the major analogue. However, the distribution of TTX analogues among tissues was not the same for the three puffer fish individuals.

Intestinal tracts and livers were again the tissues with the highest TTX concentrations for PF1 and PF2 (Figure 5A,B). Besides TTX, the specific samples demonstrated a high abundance of 11-norTTX-6(*S*)-ol and 11-deoxyTTX, followed by 5,6,11-trideoxyTTX. Regarding PF3, gonads exhibited the highest TTX levels (~162,000 μg/kg) among all tissue extracts, and it also contained 4-*epi*-5,6,11-trideoxyTTX and 5,6,11-trideoxyTTX in high abundances (~119,000 and ~988,000 μg/kg, respectively) (Figure 5C). In general, the least abundant analogues were 11-norTTX-6(*R*)-ol, 5-deoxyTTX, 6-deoxyTTX, and 4-*epi*-11-deoxyTTX, which, in fact, were not present in some tissues.

### 2.6. Application of Toxicity Equivalency Factors (TEFs) and Comparison between Techniques

**Figure 6:**
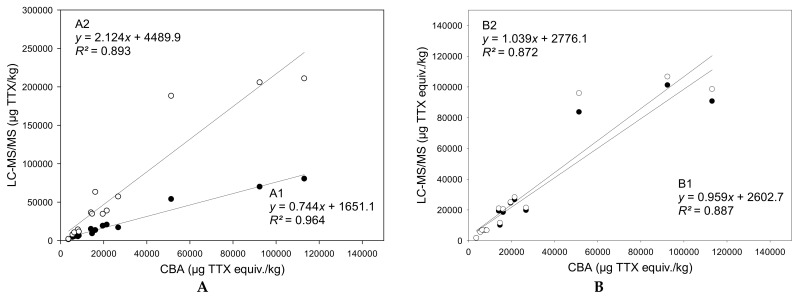
Linear regressions for the correlations between tetrodotoxin (TTX) quantifications by the cell-based assay (CBA) and LC–MS/MS analysis, before (**A**) and after (**B**) the application of toxicity equivalency factors (TEFs). (**A**): Black dots: TTX + 4-*epi*TTX; white dots: ∑all TTX analogues. (**B**): Black dots: ∑all TTX analogues with their TEFs (TEF = 0 for unknown TTX analogues); white dots: ∑all TTX analogues with their TEFs (TEF = 1 for unknown TTX analogues).

By combining the concentration data obtained by the LC-MS/MS analysis of TTX and TTX-analogues together with the respective TEF values revealed from the present study, the total toxicity was estimated for the different tissue samples of the three puffer fish individuals. A comparison of these results with the experimental measurements carried out with CBA is displayed in Figure 6. The equations in this figure show the correlations between the results obtained with CBA and different scenarios for the TTX analogues detected by LC-MS/MS. In correlation A1, only TTX and 4-*epi*TTX, which is in chemical equilibrium with TTX, were taken into account. In correlation A2, all TTX analogues were considered. Given that the PF3 gonads contained unusual high 5,6,11-trideoxyTTX and 4-*epi*-5,6,11-trideoxyTTX concentrations (~988,000 μg/kg and ~119,000 μg/kg, respectively), this datapoint was considered to be an outlier (*z*-score = 3.5) and it was excluded from all regression analyses. In correlation B1, the actual TEF values derived from the present study were applied to the corresponding TTX analogues and a TEF = 0 was assumed for the TTX analogues with unknown toxicity (in this scenario, the hypothesis is that those analogues are not toxic at all). In correlation B2, the presently obtained TEF values were applied to the corresponding TTX analogues and a TEF = 1 was assumed for the TTX analogues with unknown toxicity (in this scenario, the hypothesis is that those analogues are as toxic as TTX). It should also be pointed out that the TEFs applied in correlations B1 and B2 for 4-*epi*TTX, 11-norTTX-6(*R*)-ol, 4-*epi*-11-deoxyTTX, and 4-*epi*-5,6,11-trideoxyTTX were assumed to be those of their corresponding isomeric analogues.

When all TTX analogues were considered, the total TTX contents calculated using LC-MS/MS data were 2.1-fold higher than those obtained with CBA (y=2.124x+4489.9, *R*^2^ = 0.893). Therefore, under this scenario, LC-MS/MS analysis may overestimate the toxicity of a sample, depending on the multi-TTX profile. On the contrary, the total TTX equiv. contents provided by LC-MS/MS were 0.7-fold lower when TTX and 4-*epi*TTX were solely taken into account (y=0.744x+1651.1, *R*^2^ = 0.964). By applying the TEFs of the analogues, the correlations between LC-MS/MS and CBA TTX equiv. contents were slightly lower, but still very strong (*R*^2^ > 0.87). Nevertheless, the slopes of the respective regression lines were closer to 1, regardless of whether the TTX analogues with unknown toxic potency were assumed to have TEF = 0 (y=0.959x+2602.7, *R*^2^ = 0.887) or TEF = 1 (y=1.039x+2776.1, *R*^2^ = 0.872). This result demonstrates that the application of analogue-specific TEFs certainly improves the accuracy of toxicity prediction using LC-MS/MS-based concentration data.

## 3. Discussion

In this work, a CBA for the detection of TTXs has been optimized with the purpose of determining the TEFs of several TTX analogues and evaluating the toxicity of puffer fish samples. Results of the optimization have shown that the cell mortality (in the absence of TTX) and saturation plateau (at high TTX concentrations) depend on the concentration and ratio of O/V. Among the seven combinations tested, only three of them led to 80% of mortality in the absence of TTX (0.4/0.04, 0.35/0.035, and 0.125/0.2 O/V), a value that is considered appropriate for the subsequent detection of TTX. Nevertheless, only with 0.125/0.2 mM O/V was the saturation plateau reached (100% cell viability). These experimental conditions were chosen for subsequent experiments, since they provided an appropriate sensitivity (IC_50_ = 1.65 ng/mL) and a broader working range than the other O/V combinations. Only a few CBAs for TTXs based on the detection of cell viability with tetrazolium salt exist in the literature [44,45,46,47,48,49]. In all these studies, the optimization of O/V was an important step. Unlike other CBAs, such as those for the detection of CTXs [50] and brevetoxins [51] on their agonistic effect, CBAs for TTX are based on the antagonistic effect that they produce on cells exposed to O/V. Additionally, the sensitivity of cells to O/V and TTX depends on many factors, such as the cell line, the maintenance passage, the culture conditions, and the number of cells exposed in the assay. Not surprisingly, the other CBAs for TTXs have used different O/V combinations and have obtained different IC_50_ values, but usually in the same order of magnitude, between 2.1 and 6.6 nM [46,47,48,49,50,52]. Therefore, we can conclude that, after the optimization of the experimental parameters, we have been able to obtain a dose–response calibration curve for TTX, with the appropriate sensitivity and working range, which can be used for quantification purposes.

The scarcity or lack of TTX analogues is a problem when pursuing the establishment of TEFs. Additionally, very few isolated TTX analogues are available in sufficient amounts to develop and implement quantitative analytical methods. In fact, in LC-MS/MS analysis, concentrations of TTX analogues are usually determined assuming the same analytical response factor as for TTX (1 mole of TTX = 1 mole of TTX analogue). Therefore, in this work, the fractionation of a liver extract from an *L. sceleratus* specimen was undertaken. This fractionation process allowed the obtention of several TTX analogues with different grades of purity (Table A1). All TTX analogues of the 60 fractions were quantified with the LC-MS/MS method based on the standard curve of TTX. The presence of possible TTX mass-related interferences in the different puffer fish extracts was also evaluated. A signal was recorded in the chromatograms from the digestive and liver of PF1 and PF2 for both 5,6,11-trideoxyTTX transitions monitored at min 4.5 (RT) (Figure 2). Nonetheless, the ion ratio calculation ruled it out as a trideoxyTTX analogue. Due to the number of TTX analogues present in PF2 liver tissue, and the inherent characteristics of the HILIC chromatographic method, a 100% grade of purity of TTX analogues was only achieved in three pooled fractions, which contained 5,6,11-trideoxyTTX. However, the concentration of this analogue in these fractions was not enough to conduct the proposed assays (CBAs). Nevertheless, fraction 23, with 93% purity of 5,6,11-trideoxyTTX, was used for that purpose.

Knowledge of TEF_S_ of TTX analogues is crucial to translate TTX analogue contents obtained from instrumental analysis techniques, such as LC-MS/MS, into global toxicity estimations expressed as TTX equivalents. In the present work, we used the CBA to establish the TEFs of four TTX analogues (5,11-dideoxyTTX, 11-nor-TTX-6(*S*)-ol, 11-deoxyTTX, and 5,6,11-trideoxyTTX) that have been obtained and purified from a liver of an *L. sceleratus* individual. As expected from the literature [47,48,49], the calibration curves revealed that the four analogues are less toxic than the parent TTX. The IC_50_ of 5,11-dideoxyTTX, 11-norTTX, 11-deoxyTTX, and 5,6,11-trideoxyTTX were, respectively, 1.3-, 2.5-, 7.2-, and 90-fold higher than that of TTX. Therefore, the toxicity trend was TTX > 5,11-dideoxyTTX > 11-norTTX-6(*S*)-ol > 11-deoxyTTX > 5,6,11-trideoxyTTX. The information available on the relative toxicity of TTX analogues in the literature is rather scarce, which makes a comparison with other studies difficult. Some TEFs have been established with animal bioassays with mice after intraperitoneal injection and some of them with Neuro-2a CBA (Table 3). TEFs obtained for synthetic TTX analogues, not occurring naturally, were not included in Table 4.

Differences in TEFs between methods, and even within the same method, are not surprising, considering the different detection principles, the different experimental parameter of the methods, the sensitivity of the cells, and the different ways used to calculate the TEF determination. In fact, other authors also claim these differences among methods [42,61]. In the CBA, the effect on cells is evaluated, while, in the MBA, the effect is observed in the whole animal. As previously mentioned, the cell line, maintenance passage, number of cells in the assay, and O/V ratio and concentration may also cause differences. In the case of CBAs, TEFs are usually calculated as the ratio of the IC_50_ value of the TTX standard to the IC_50_ value of the TTX analogue. However, the calculation of TEFs from MBA results can be presented in different ways, using parameters like minimum lethal dose (MLD), lethal dose 50% (LD_50_), lethal dose 99% (LD_99_), IC_50_ (all these parameters expressed in μg/kg bw), or toxic potency (MU/mg compound) [28]. The differences in the number of mice used may also influence the results. Other parameters that may have an influence on the calculation of TEFs are the purity of the TTX analogues, and the interferences from unknown toxins or other compounds from the matrix, if any. Although difficult, we have tried to compare our results with those found in the literature (except for 5,11-dideoxyTTX, since its TEF had not been determined before this study). In general terms, the toxicity trend TTX > 11-norTTX-6(*S*)-ol > 11-deoxyTTX > 5,6,11-trideoxyTTX established in our work was also observed in the values available in the literature, regardless of the method. Our TEFs of 5,6,11-trideoxyTTX and 11-deoxyTTX were similar to those found in mice [60]. However, the TEF obtained in our study for 11-deoxyTTX was ~9-fold higher than those obtained with CBA [47,48]. For 11-norTTX-6(*S*)-ol, the TEF established in this study was ~2-fold higher than in MBA [58]. The low toxicity of 5,6,11-trideoxyTTX has also been reported in many studies [24,62,63]. In fact, several authors have observed that hydroxyl groups have an important role in the toxicity of a TTX analogue [47,48,49,64,65,66,67,68]. These hydroxyl groups are involved in the Na_v_ blocking activity of TTX analogues due to the formation of hydrogen bonds. Therefore, TTX analogues with fewer hydroxyl groups, compared with TTX, could have a lower affinity for the binding to the sodium channel, which may lead to a decrease in their toxicity. In our work, 5,6,11-trideoxyTTX was much less toxic than 5,11-dideoxyTTX, the only difference between them at a structural level being the hydroxyl group in C-6 (absent in 5,6,11-trideoxyTTX). The differences in TEFs found in the literature make their choice in monitoring programs difficult. The limited description of the studies and the different ways in which the toxicity is expressed make it so that the uncertainties associated with estimation of these relative potencies are very high. A comparison between methods, protocol harmonization, and interlaboratory validations would certainly contribute to clarifying and better understanding which TEFs to use and why.

The TTX levels in the muscle, skin, liver, intestinal tract, and gonads of the three puffer fish individuals, caught on the Crete coasts between March and May 2019, were investigated using two different techniques, CBA and LC-MS/MS. The CBA provides a global toxicological response from TTX and TTX analogues, whereas LC-MS/MS analysis determines individual TTX and TTX analogue contents. Ideally, in the analysis of samples both in research and monitoring programs, a combination of techniques is desired, as they provide complementary information. However, due to budgetary reasons, this is not always possible. In this work, the CBA indicated that the intestinal tract and liver were the tissues with the highest TTX concentrations, followed by the skin and muscle. Nevertheless, all the tissues contained TTX at levels higher than the dose considered safe for human consumption according to Japanese legislation (2 mg TTX equiv./kg). Gonads of PF1 and PF2 exhibited the lowest TTX contents but, nonetheless, gonads of PF3 exhibited the highest TTX concentration among all tissue extracts, which was more than 100-fold above the Japanese acceptability criterion. Tetrodotoxin levels found in *L. sceleratus* with CBA were in the range of those obtained from previous studies: 50–58,440, 100–35,050, 90–1,380,800, 70–478,430, and 170–8,248,510 μg TTX equiv./kg for muscle, skin, liver, intestinal tract, and gonads, respectively (Table 5). The normally higher TTX content in gonads, intestinal tract, and liver compared to skin and muscle have also been observed in other studies [14,15,16,17,18,19,21]. Puffer fish 3 was a female individual caught in March. Although the number of puffer fish individuals in this study is very limited and statistical conclusions cannot be drawn, the higher TTX levels in females compared to males, observed in this study, have also been previously reported [14,21,69,70]. The high TTX values found in the gonads of this female specimen are in agreement with previous studies [14,15,16,17,21,69,70,71]. These studies [14,21,69,70,71] report that, during the spawning period, TTX is transferred from the liver to the ovaries, resulting in higher TTX levels, which will then be transferred to puffer fish eggs, and which may act as a mechanism of defense (both for the eggs and the larvae) against the risk of predation. Furthermore, other studies [72,73] justify the differences in TTX contents between female and male puffer fish during the maturation/spawning period by the role of TTX as a male-attracting pheromone. Finally, other studies reported the relationship between the high toxicity of *L. sceleratus* and the size of the specimen [73], which may explain the higher toxicity found in PF3 in comparison with PF1 and PF2.

Tetrodotoxin (TTX) was the major analogue found in almost all tissues of the three *L. sceleratus* specimens analyzed in this work, with abundances between 20 and 70% depending on the tissue and the specimen. In PF1 and PF2, TTX was followed by 11-norTTX-6(*S*)-ol (between 9 and 31%) and 11-deoxyTTX (between 4 and 24%); the 5,6,11-trideoxyTTX analogue was also abundant in the skin of both specimens (28 and 40% for PF1 and PF2, respectively). In PF3, the toxin profile was very different, TTX being followed by 5,6,11-trideoxyTTX (between 33 and 50%) and 4-*epi*-5,6,11-trideoxyTTX (between 5 and 22%). Christidis and collaborators [21] found that TTX and 11-deoxyTTX were among the most abundant TTX analogues in all tissues (between 41 and 64%, and between 23 and 30%, respectively), and that 5,6,11-trideoxyTTX was present in high concentrations in some samples but completely absent in others. Bane and colleagues [74] also identified TTX, 11-deoxyTTX, 5,6,11-trideoxyTTX, as well as 11-norTTX-6(*S*)-ol as the most abundant, the relative percentages depending on the specimen and the tissue. Rodriguez [15] and Rambla-Alegre [19] reported that 5,6,11-trideoxyTTX was the major TTX analogue detected. The high abundance of 5,6,11-trideoxyTTX in the ovaries of puffer fish has been attributed to its use as an odorant or as an attractive sex pheromone for toxic puffer fish [67]. The differences between studies may be attributed to the number of specimens analyzed, the season and region in which the individuals are caught, their maturity stage, and the diet. As previously mentioned, the number of puffer fish specimens in this study is limited, since the purpose was to set up a CBA for TTXs, to elucidate the TEFs for several TTX analogues, and to demonstrate their applicability in the analysis of puffer fish. Therefore, general trends and a comparison with other works must be cautiously interpreted.

The application of TEFs to the individual TTX analogue concentrations obtained with LC-MS/MS is necessary for estimating the overall toxicity of a sample. The TTX analogues may contribute to the toxicity of the *L. sceleratus* samples to a greater or lesser extent depending on their concentration and their toxic potency. When the contribution of TTX analogues detected with LC-MS/MS in the different tissues of the three puffer fish individuals was not considered, the TTX contents were 0.7-fold lower than those provided by CBA (Figure 6A). When, on the contrary, the TTX analogues were considered, LC-MS/MS overestimated the TTX contents by 2.1 times. As mentioned above, the PF3 gonad datapoint was removed from the regression analysis. The reason was the unusual high 5,6,11-trideoxyTTX and 4-*epi*-5,6,11-trideoxyTTX concentrations; its inclusion in the regression A2 causes the overestimation value to change from 2.1 to 5.1 times. This effect is not noticed when other datapoints are deleted. The application of the obtained TEFs to the TTX analogues (when available from this work) resulted in a slight decrease of the coefficients of determination but improved the slopes, making them closer to 1 and, therefore, indicating that, in general, similar data are obtained with both techniques (Figure 6B). However, it is necessary to mention that, when observing particular data points, discrepancies may be observed. For example, for the liver of PF2, LC-MS/MS analysis still overestimates the results from CBA by a factor of 2. The fact that there are no significant differences when the TTX analogues with unknown TEFs were considered as non-toxic (TEF = 0) or as toxic as TTX (TEF = 1) can be explained by the small abundance of these TTX analogues in relation to the total TTX content in the samples. In fact, as previously mentioned, TTX was generally the most abundant analogue found in these samples and, therefore, had a lot of weight in the correlations. Nevertheless, the improvement of the slopes of the regression equations after the application of TEFs clearly demonstrates the applicability and the importance of TEFs in obtaining useful toxicological estimations from LC-MS/MS analysis.

The establishment of TEFs is also very important for gaining knowledge of the toxicity of TTX analogues. Therefore, TEFs may help official monitoring programs to prioritize the research and investigation on those analogues that are more toxic and that may cause poisoning in humans, rather than those with lower toxicity. This would contribute to properly estimating the toxicity of samples before they enter the commercial chain and reach consumers. Additionally, TEFs may also be useful in projecting the overall toxicity of samples with multi-TTX profiles in the ecosystems. The eventual metabolization of TTXs throughout the food webs may lead to changes in the TTX analogue profile. Therefore, the TEFs are useful for understanding how the toxicity of the samples may evolve along the food webs.

## 4. Materials and Methods

### 4.1. Puffer Fish Samples and Tetrodotoxin (TTX) Standard

Three puffer fish specimens, two males (PF1, PF2) and one female (PF3), morphologically identified as *Lagocephalus sceleratus*, were captured with trammel net on Crete coasts of the Libyan Sea (Greece, E Mediterranean Sea) in May (PF1, PF2) and March 2019 (PF3). The three fishes were dissected and separated into muscle, skin, liver, intestinal tract, and gonads, and the tissues were stored at −20 °C until required.

TTX standard was purchased from Abcam plc (Cambridge, UK) and the TTX standard solution was prepared at 1 mg/mL in 1% acetic acid.

### 4.2. Extraction of Tetrodotoxins (TTXs) from Puffer Fish Samples

The extraction of TTXs from the different puffer fish tissues was performed according to Reverté and co-authors [32] with slight modifications. Briefly, 10 g of puffer fish tissues were weighed into tubes and homogenized with a Ultraturrax blender at full speed. In each tube, 25 mL of 0.1% acetic acid was added and vortexed for 2 min at 2500 rpm. Then, tubes were placed in a boiling water bath for 10 min with occasional stirring. Then, tubes were cooled down and centrifuged at 2500 rpm for 5 min (at 4 °C) and the supernatants were transferred to new tubes. A second extraction was performed on the pellets with 20 mL of 0.1% acetic acid. The two supernatants were pooled, and the final volume was set to 50 mL with 0.1% acetic acid. In the case of liver samples, an additional liquid−liquid partition of the crude extract with hexane (1:1) was required to remove fats. For the CBA analysis, the extracts were filtered using 0.45 μm nylon syringe filters. For LC-MS/MS analysis, extracts were also filtered through 3000 Da molecular sieve filters and 0.2 μm nylon syringe filters. The final extracts contained 200 mg equiv. of puffer fish tissue/mL and were stored at −20 °C until required.

### 4.3. Isolation and Purification of Tetrodotoxin (TTX) Analogues

Tetrodotoxin analogues were obtained from the liver of PF2 (extraction from 30 g). This extract was reduced to 5 mL under reduced pressure at 70 °C using a rotary evaporator (Büchi Rotavapor R-200 coupled to a Büchi Heating Bath B-490) and mixed with 5 mL of acetonitrile. The fractionation protocol was based on a previous work [32]. A 600-MS LC pump system controller (Waters Corp. Milford, MA, USA) coupled to a photodiode array detector (PDA) 996 (Waters Corp.) and a fraction collector FRAC-100 (Pharmacia Biotech, Uppsala, Sweden) were used. Empower software (Waters Corp.) was used for the instrument control. Hydrophilic interaction chromatography (HILIC) fractionation was conducted at room temperature and 10 mL/min on a prep-LC column Luna HILIC AXIA (250 mm × 21.2 mm, 5 μm particle size) purchased from Phenomenex (Torrance, CA, USA). Binary gradient elution was performed with Milli-Q water (mobile phase A) and acetonitrile/water (90/10, *v*/*v*, mobile phase B) (HPLC grade), both containing 30 mM ammonium acetate at pH 5.8. The gradient program started at 100% B, and it was kept isocratic for 5 min; then, it was decreased down to 95% B at min 35. Afterwards, it was decreased down to 82.5% B at min 80, was held at isocratic for 5 min, and was returned to initial conditions at min 90. A total of 10 runs were performed as follows: for each run, 1 mL of concentrated extract was manually loaded on a 1 mL loop, and once the chromatographic run started, 10 mL fractions were automatically collected every minute. At the end, the same fractions from each run were pooled (total of 100 mL) in 125 mL bottles. Fractions were stored at −20 °C until required.

### 4.4. Liquid Chromatography-Tandem Mass Spectrometry (LC-MS/MS) Analysis

Liquid chromatography-tandem mass spectrometry was used for the analysis of the fractions and the puffer fish. When necessary, samples were diluted to address proper TTX and TTX analogue quantification. We checked in every batch the quality control criteria stated by the TTX EURLMB SOP regarding limits of quantification (LOQs) and linearity [75]. The coefficients of determination (*R*^2^) of the calibration curves had to be higher than 0.98 to ensure linearity and the deviation of the slopes between consecutive calibration curves had to be lower than 25% to be considered acceptable. Eight-level calibration doses between 0.078–10 ng/mL (0.39–50 μg TTX/kg) showed good intra-batch performance and linear adjustment (*R*^2^) ≥ 0.998; the deviation of the slopes between consecutive scans was 16%, and the LOQ was 0.39 μg TTX/kg.

For LC-MS/MS analyses, a tandem quadrupole mass spectrometer (Xevo^TM^ TQ-XS, Waters Corporation, Milford, MA, USA) coupled to a UPLC binary pump system (Acquity UPLC I-plus-Class, Waters Corporation, Milford, MA, USA) was used. A nitrogen generator NM20Z (Peak scientific, Renfrewshire, Scotland, UK) supplied all operation gases. Instrument control, acquisition, and data analysis were powered by MassLynx V4.2 (Waters Corporation, Milford, MA, USA) and TargetLynx XS software (Waters Corporation, Milford, MA, USA).

Analytical separations were performed at 60 °C on a Acquity UPLC^®^ BEH amide column (150 mm × 2.1 mm, 1.7 μm particle size) from Waters Corp. (Milford, MA, USA) and flow rate ranged from 0.4 to 0.8 mL/min. Liquid chromatography analysis was performed according to the EURLMB Standard Operating Procedures (SOPs) for the determination of TTX by HILIC-MS/MS [70]. Briefly, a binary gradient elution was programmed with water as mobile phase A containing 0.015% of formic acid and 0.06% of ammonium hydroxide LC-MS additive (25% as NH_3_), and as mobile phase B acetonitrile/water (70/30, *v*/*v*) containing 0.01% of formic acid. The 11 min gradient run time method was used starting with 98% B for 5 min at 0.4 mL/min, then it was decreased down to 50% B for 7.5 min, held at 50% B until min 9, and then it was increased to 95% of B at min 9.5 at 0.5 mL/min, and then set back to 98% of B at min 9.8 at 0.8 mL/min until min 10.6, when, finally, initial conditions were reached at 0.4 mL/min, which were held at the starting conditions for column equilibration until a total run cycle of 11 min. Mobile phase eluting from the column was diverted to waste for the first 1 min of analysis. Methanol was used as solvent to wash the autosampler needle. Injection volume was 2 μL, and the autosampler was cooled at 8 °C. Extracts were analyzed with the mass spectrometer operating in positive polarity, using the parameters optimized for the TTX standard in ESI (+) mode (source temperature: 150 °C, desolvation temperature: 600 °C, gas flow desolvation: 1000 L/hour, cone gas flow: 150 L/hour, and nebulizer gas flow: 7 bar). Multiple reaction monitoring (MRM) transitions were monitored for the following TTX analogues (precursor ion > product ion 1 (collision energy, CE)/product ion 2 (CE)): 320.1 > 302.1 (20 V)/162.2 (40 V) for TTX and 4-*epi*TTX, 302.1 > 256.1 (30 V)/162.2 (30 V)/284.1 (30 V) for 4,9-anhydroTTX, 304.1 > 286.1 (30 V)/ 162.2 (30 V)/176.1 (30 V) for monodeoxyTTX analogues, 288.1 > 270.1 (20 V)/224.1 (20 V)/ 162.1 (20 V) for dideoxyTTX analogues, 272.1 > 254.1 (30 V)/162.2 (30 V) for trideoxyTTX analogues, and 290.1 > 272.1 (30 V)/162.2 (30 V) for 11-norTTX 6(*S*)-ol and 11-norTTX-6(*R*)-ol. Additionally, arginine and hidroxyarginine were monitored with 176.0 > 60 (30 V)/70 (30 V) and 192.0 > 68 (30 V)/86 (30 V), respectively. Dwell time was 14–16 ms for all transitions. At least two MRM transitions were monitored, and identification was supported by toxin retention time and MRM ion ratios. Quantification of TTX analogues was performed against TTX, assuming equimolar responses, given the equivalent structural fragmentation in MRM transitions. Measurements were performed in duplicate.

### 4.5. Cell Maintenance and Cell-Based Assay (CBA)

RPMI-1640 medium, fetal bovine serum (FBS), penicillin, streptomycin, sodium pyruvate, ouabain, veratridine, phosphate buffered saline (PBS), and thiazolyl blue tetrazolium bromide (MTT) were purchased from Merck KGaA (Gernsheim, Germany).

Neuroblastoma murine (Neuro-2a) cells were purchased from ATCC LGC standards (Manassas, VA, USA) and maintained in RPMI-1640 medium, supplemented with 10% FBS, 1% penicillin–streptomycin and 1% sodium pyruvate, in an incubator (BINDER GmbH, Tuttlingen, Germany) at 37 °C in 5% CO_2_ humid atmosphere.

For the CBA, Neuro-2a cells were trypsinized and suspended in culture medium (the same as for maintenance but with 5% FBS instead of 10% FBS). Then, Neuro-2a cells were seeded in a 96-well microplate at an approximate density of 35,000 cells/well in 200 μL of culture medium for 24 h at 37 °C in 5% CO_2_ humid atmosphere. Prior to exposure to TTX standard solution, TTX analogue solution, or puffer fish tissue extract, some Neuro-2a cells were pre-treated with 20 μL of an O and V mixture in PBS at final concentrations of 0.125 and 0.2 mM, respectively (0.5/0.05, 0.45/0.045, 0.4/0.04, 0.35/0.035, 0.3/0.03 0.2/0.1, and 0.125/0.2 mM for the optimization). Tetrodotoxin standard solution, TTX analogue solution, or puffer fish tissue extract were dried under an N_2_ stream at 40 °C using a TurboVap evaporator (Zymark corp., Hopkinton, Massachusetts), reconstituted in culture medium, and serially diluted, and 10 μL was added to the wells with and without O/V pre-treatment. After 24 h, cell viability was measured using the MTT assay [44]. Absorbance at 570 nm was measured with a Synergy LX microplate reader from BioTek (Agilent Technologies, Inc., Santa Clara, CA, USA). Measurements were performed in triplicate.

## 5. Conclusions

In this study, the optimization of a CBA for TTX detection has been successfully achieved. The applicability of this assay to the analysis of the different tissues of three *L. sceleratus* specimens has been demonstrated, confirming the presence of TTX equiv. in all samples. The measured toxicity of all samples exceeded the Japanese safety level of TTX in puffer fish tissues. LC-MS/MS analysis, as a confirmatory method to identify the presence of TTX and its analogues, has been performed. The obtained results demonstrate a multi-toxin profile for all samples analyzed, with TTX being the major analogue detected almost in all tissues of the three *L. sceleratus* specimens. Another remarkable result was the high concentrations of TTX found in the ovaries of PF3 compared to the other tissues analyzed. Moreover, we successfully purified and isolated several TTX analogues from a liver extract. The optimized CBA was applied to four of these TTX analogues (5,11-dideoxyTTX, 11-norTTX-6(*S*)-ol, 11-deoxyTTX, and 5,6,11-trideoxyTTX). The TEFs for these TTX analogues have been established, the one for 5,11-dideoxyTTX being the first reported in the literature. All TTX analogues have been observed to be less toxic than TTX. Then, these TEFs have been applied to the results obtained with LC-MS/MS analysis. The improvement of the results after the application of TEFs evidences the importance and the utility of TEFs in the estimation of the total toxicity of a sample and, therefore, in risk assessment.

## Figures and Tables

**Table 1:** Cell viability, half-maximal inhibitory concentration (IC_50_) values, and saturation plateaus for tetrodotoxin (TTX) in presence of different ouabain (O)/veratridine (V) combinations as determined by Neuro-2a cell-based assay (CBA).

O/V Combinations (mM)	Cell Viability (%)	IC_50_ (ng/mL)	Saturation Plateau (%)
0.5/0.05	28 ± 2	8.65	57 ± 1
0.45/0.045	29 ± 2	3.55	63 ± 2
0.4/0.04	21 ± 2	1.07	78 ± 3
0.35/0.035	20 ± 2	1.67	72 ± 3
0.3/0.03	50 ± 2	2.69	68 ± 2
0.2/0.1	38 ± 1	2.37	86 ± 4
0.125/0.2	20 ± 1	1.65	102 ± 3

**Table 2:** Half-maximal inhibitory concentration (IC_50_) values and toxicity equivalency factors (TEFs) for tetrodotoxin (TTX) and TTX analogues as determined by Neuro-2a cell-based assay (CBA).

TTX or TTX Analogue	IC_50_ (ng/mL)	TEF
TTX	1.65	1
5,11-dideoxyTTX	2.20	0.750
11-norTTX-6(*S*)-ol	4.08	0.404
11-deoxyTTX	11.88	0.139
5,6,11-trideoxyTTX	147.78	0.011

**Table 3:** General description of the three puffer fish individuals.

	Size (cm)	Weight (g)	Sex	Fishing Date	Fishing Area	Depth (m)
PF1	53.5	1840	Male	14/5/2019	Ierapetra	18
PF2	53.9	1700	Male	14/5/2019	Ierapetra	18
PF3	59.3	2200	Female	20/3/2019	Agia Galini	35

**Table 4:** Toxicity equivalency factors (TEFs) for tetrodotoxin (TTX) analogues evaluated with mouse bioassay (MBA) and Neuro-2a cell-based assay (CBA) from different studies, including the present one (in bold).

TTX Analogue	TEF	Detection Method	Reference
4-*epi*TTX	0.16	MBA	[53]
6-*epi*TTX	0.17	MBA	[54]
5-deoxyTTX	0.01	MBA	[55]
6-deoxyTTX	0.32	CBA	[47]
**11-deoxyTTX**	0.14	MBA	[54]
	0.016	CBA	[47]
	0.017	CBA	[48]
	**0.139**	**CBA**	**[This study]**
11-oxoTTX	0.75	MBA	[56]
	1.621	CBA	[49]
11-norTTX-6(*R*)-ol	0.17	MBA	[57]
**11-norTTX-6(*S*)-ol**	0.19	MBA	[58]
	**0.404**	**CBA**	**[This study]**
**5,11-dideoxyTTX**	**0.750**	**CBA**	**[This study]**
6,11-dideoxyTTX	0.02	MBA	[59]
	0.005	CBA	[47]
4,9-anhydroTTX	0.02	MBA	[53]
**4,4a-anhydroTTX**	0.0014	CBA	[49]
**5,6,11-trideoxyTTX**	0.01	MBA	[60]
	**0.011**	**CBA**	**[This study]**

**Table 5:** Tetrodotoxin (TTX) concentrations (μg TTX/kg) in different tissues of *L. sceleratus* from different studies, including the present one (in bold).

Region	Muscle	Skin	Liver	Intestinal tract	Gonads	Method	Reference
Rhodes Island, Greece	<1100–10,160	<1100–6630	16120–87,530	6310–177,650	17,050–239,320	MBA	[18]
Rhodes Island, Greece	<320-58,440	<320–33,340	<320–1,380,800	<320–478,430	470–8248,510	LC-ESI-CID-MS/MS	[15]
Northeastern Mediterranean	ND–2830	130–3430	ND–46,180	70–7150	430–52,070	LC-MS/MS	[16]
Western Mediterranean	1010	1650	3080	-	25,590	LC-MS/MS	[20]
	980	2080	5360	-	25,220	LC-HRMS	
	2530	3500	28,300	-	33,550	mELISA	
North Aegean Sea (Greece)	1395–2878	2588–2780	2882 *		-	Immunosensor	[13]
	478–2077	1188–1239	733 *		-	LC-HRMS	
	1520–2327	2773–3137	10,834 *		-	Immunoassay	
Cretan and Libyan Sea	50–41470	170–35,050	90–312,950	-	430–535,780	LC-MS/MS	[21]
Northern Cyprus Sea	210–8320	160–6540	110–13,480	290–11,740	320–12,870	Immunoassay	[14]
Eastern Mediterranean	100–3420	100–3300	120–25,400	-	170–80,000	LC-MS/MS	[19]
**Libyan Sea**	**5558–14,091**	**8032–16,116**	**21,453–51,350**	**19,584–113,127**	**3657–228,881**	**CBA**	**[This study]**
	**7640–36,486**	**14,251–63,178**	**38,917–188,240**	**34,646–210,873**	**2129–1,324,439**	**LC-MS/MS**	

* In internal organs of juvenile *L. sceleratus* (liver and intestinal tract). ND: not detected. LC-ESI-CID-MS/MS = liquid chromatography electrospray ionization tandem mass spectrometry. LC-HRMS = LC coupled to high-resolution mass spectrometry. mELISA = maleimide-based enzyme-linked immunosorbent assay.

## Data Availability

The data are contained within the article are available upon request.

## Appendix A

**Table A1:** Concentration and grade of purity of tetrodotoxin (TTX) analogues in the fractions (only TTX analogues used for the determination of the corresponding TEFs) (used fractions in bold).

	TTX analogue (ng/mL and %)							
Fraction	5,6,11-trideoxyTTX		5,11-dideoxyTTX		11-deoxyTTX		11-norTTX-6(*S*)-ol	
16	0.06	100.0%						
17	0.44	100.0%						
18	9.24	100.0%						
19	13.34	67.7%						
20	30.34	25%						
21	19.93	38.1%						
22	77.21	80.2%						
**23**	**83.26**	**92.9%**						
24	67.30	94.1%						
25	32.87	90.4%	0.55	1.5%				
26	16.21	82.4%	1.60	8.1%				
28	15.12	77.4%	3.83	19.6%				
29	1.33	41.2%	1.79	55.2%				
30	0.85	4.4%	18.57	95.2%				
**31**	0.09	1.5%	**5.85**	**98.2%**				
**32**	0.12	1.4%	**8.49**	**98.2%**				
33	0.03	0.4%	8.35	99.4%				
34	0.04	0.4%	9.35	93.9%				
35	0.05	1.8%	1.50	57.0%				
39			0.09	0.8%	0.22	1.8%		
40			0.09	0.3%	0.41	1.5%		
41					0.76	2.5%	0.04	0.1%
42					2.67	9.4%	0.33	1.2%
46					8.69	66.2%	0.03	0.3%
47					15.98	77.4%	0.10	0.5%
48					55.23	92.5%	0.31	0.5%
**49**					**69.90**	**94.0%**	0.49	0.7%
50					95.52	94.0%	0.82	0.8%
51					136.13	94.0%	1.72	1.2%
52					93.58	88.3%	4.51	4.3%
53					24.53	62.6%	5.01	12.8%
54					15.51	46.2%	6.33	18.9%
56					9.61	8.5%	91.46	80.7%
**57**					6.83	5.1%	**113.52**	**85.4%**
58					4.81	2.8%	151.00	89.5%
59					2.95	1.4%	111.47	51.7%
60					2.12	3.8%	41.05	72.9%
